# Community perceptions and mental burden among (former) residents at Europe’s largest lignite mine in Western Germany: a cross-sectional study

**DOI:** 10.1038/s41598-025-92834-8

**Published:** 2025-03-14

**Authors:** Emma Holtermann, Theresa Krüger, Thomas Kraus, Andrea Kaifie

**Affiliations:** 1 Institute for Occupational, Social, and Environmental Medicine, Medical Faculty, RWTH Aachen University, Pauwelsstrasse 30, 52074 Aachen, Germany; 2 Institute for Occupational, Social, and Environmental Medicine, FAU Erlangen-Nürnberg, Henkestraße 9&11, 91054 Erlangen, Germany

**Keywords:** Resettlement, Open pit mining, Solastalgia, Environmental distress, Mental health, Psychology, Environmental impact

## Abstract

During the many years that coal has been used for energy production, most studies have focused on the global environmental impacts such as climate change or, when addressing health, rather on conditions of open pit coal miners. This study aims to assess the mental health impacts of lignite (brown coal) mining on communities living in proximity to an open pit mine, including resettlers. A questionnaire-based survey was used with items including socioeconomic effects of open pit mining and resettlement, environmental burden, attitudes, as well as scales for depressive and somatic symptoms. Data included *n* = 100 respondents from resettled villages and *n* = 235 participants from pit edge villages nearby Europe’s largest open pit mine Hambach in Western Germany. Both groups reported up to twice as high levels of depressive and somatic symptoms compared to the general population. Over 16.0% of all surveyed stated moderate-to-severe depressive symptoms, while 40.0% of respondents from pit edge villages self-reported moderate-to-severe somatic symptom levels (vs. 23.7% in resettled villages). Three out of four respondents from pit edge villages felt often to nearly always affected by dust followed by noise (41.9%). These results suggest that a changing home environment and residential proximity to an open pit mine could affect the mental health, leading to psychological strain. Further studies on the psychosocial impact of environmental change and loss of home are needed.

**Supplementary Information:**

The online version contains supplementary material available at 10.1038/s41598-025-92834-8.

## Introduction

### Fossil fuels at a global to National scale

At the 26th Climate Change Conference in Glasgow on October 31 - November 13, 2021, the participating parties agreed to a “phase-down of unabated coal power” and “phase-out inefficient fossil fuel subsidies”, in order to accelerate their climate protection efforts^1^. 35 countries and four institutions, including Germany, signed a joint commitment on ‘International Public Support for the Clean Energy Transition’, aiming to end new direct public support for the international fossil fuel energy sector by the end of 2022^2^. Lignite is the main source of energy among the fossil fuels used to supply electricity in Germany^3^. In 2020, Germany’s coal phase-out law stipulated that no more electricity may be generated by coal after the end of 2038; according to the current coalition agreement even until 2030^4,5^. This Act^4^ is an important measure to reduce greenhouse gas emissions, mitigate climate disasters and diminish global heating, while it also ceases local coal extraction-related pollution and the need for further resettlements.

### Research trend of open pit mining

Coal production and combustion lead to various hitherto insufficiently examined problems at a regional scale. When dealing with health, only workers in open pit mines were usually the subject of research^6^. In the USA, for example, surveillance programs (e.g., the Coal Workers’ Health Surveillance Program) were implemented to be able to record longer-term effects on the health of open pit miners^7,8^. However, open pit mining does not only affect employees. Residents living near those mines can also be impacted by environmental pollution (e.g., coal dust or noise) and resulting health conditions^9^. So far, those communities are not sufficiently represented in the research landscape^10^. Residential studies mainly focused on noise and its potential (mental) health effects, e.g. relationship between noise exposure and depressive disorders^11–13^ or the dimension of noise disturbance^14,15^. In Queensland, research was done on the mental health conditions of residents in coal mining regions, what support services they would find helpful, and how these have to be expanded^16,17^.

Consequences residents are facing can also include extensive ecosystem^18,19^ and landscape changes^20^, as well as impairment of local agriculture^21^. Van Haaften described how ‘an environment that starts to erode will induce psychological consequences such as stress and marginalization’^22,23^.

So far only one study in Germany quantitatively assessed potential mental health effects associated with open pit mining focusing on the Garzweiler open pit mine in Western Germany^14^. To generate additional and more comprehensive results, it appears interesting to examine further locations, with a different local and historical context.

Hambach is Europe’s largest open pit mine, covering an area of approximately 4300 hectares^24^. The mine is located in Western Germany (Rhenish region) where in 2021 almost 50% of Germany’s lignite was extracted^25^. Due to the statutory coal phase-out in Germany, the Hambach open pit mine will be closed earlier than planned and no more residents are being resettled nowadays. Current plans envisage the conversion of the open pit mine into a lake while recultivation measures are to be continued^26^. Due to the Hambach open pit mine, large parts of the Hambach Forest had to be cleared. This led to a considerable (international) media attention, due to many demonstrations and longstanding presence of environmental activists in the immediate vicinity of the open pit^27^. As part of the recultivation measures of the spoil heap in the north of the open pit mine Hambach, the Sophienhöhe was created for forestry restoration of the destroyed nature^28^.

### Research objectives and study aim

While it is suggested that environmental degradation has an impact on people’s mental health^29–31^, the role of the familiar environment is particularly important. A changing home environment can also lead to a feeling of distress known under the technical term solastalgia^32^. People feel solastalgia when their sense of place is negatively affected and “manifests in a feeling of dislocation” from their home environment^33^. Reasons can be rather natural (e.g. climate change) but also artificial factors like war, gentrification, or mining^33^. The concept of Solastalgia itself is rooted in a study on a huge mining region in Australia^34^.

So far, some studies addressed the different components of change associated with open pit mining, such as environmental degradation, resettlement and altered social structures.

Our study aims to assess the mental health burden in residents living close to the open pit mine Hambach compared to already resettled people who previously lived close to the Hambach mining area. In addition, study population specific stressors, such as environmental hazards caused by mining for pit edge villagers as well as stressors caused by the resettlement process were determined.

## Materials and methods

### Study population and data gathering

The cross-sectional survey focused on residents, older than 17 years, who lived less than 7 km next to the open pit mine Hambach (pit edge villagers) or who resided there prior to their resettlement (new villages). Both study populations were determined since it is assumed that mining has a different impact on already resettled persons in comparison to residents next to a coal mine.

In April and May 2022, data was collected mainly in three pit edge villages (Buir, Elsdorf, Niederzier-Ellen) and three new villages (Neu-Etzweiler, Morschenich-Neu, Manheim-Neu; meaning newly built settlements for people relocated because of open pit mining), both shownFig. 1. In general, the new villages were located much further away from the mining site (on average 3.8 km distance compared to 2 km for pit edge villages), assuming a lower mining-caused environmental exposure. Paper-based and online questionnaires were used to reach people of different age, media use and place of residence.

In pit edge villages as well as new villages, 600 questionnaires were distributed by drop off at home, including stamped envelopes for return. A link to the online questionnaire with general information about the study was published via social media (Facebook, Twitter) with the support of local citizens’ initiatives as well as in a local newspaper. Ethical permission for data collection was given from the local Human Research Ethics Committee of the RWTH Aachen University Faculty of Medicine (EK094-22, March 2022).

All participants gave their informed consent to anonymously participate in the study before filling out the questionnaire. It was declared that there were no negative consequences for not filling out the questionnaire. A contact person for psychological support for respondents was named.

**Fig. 1:**
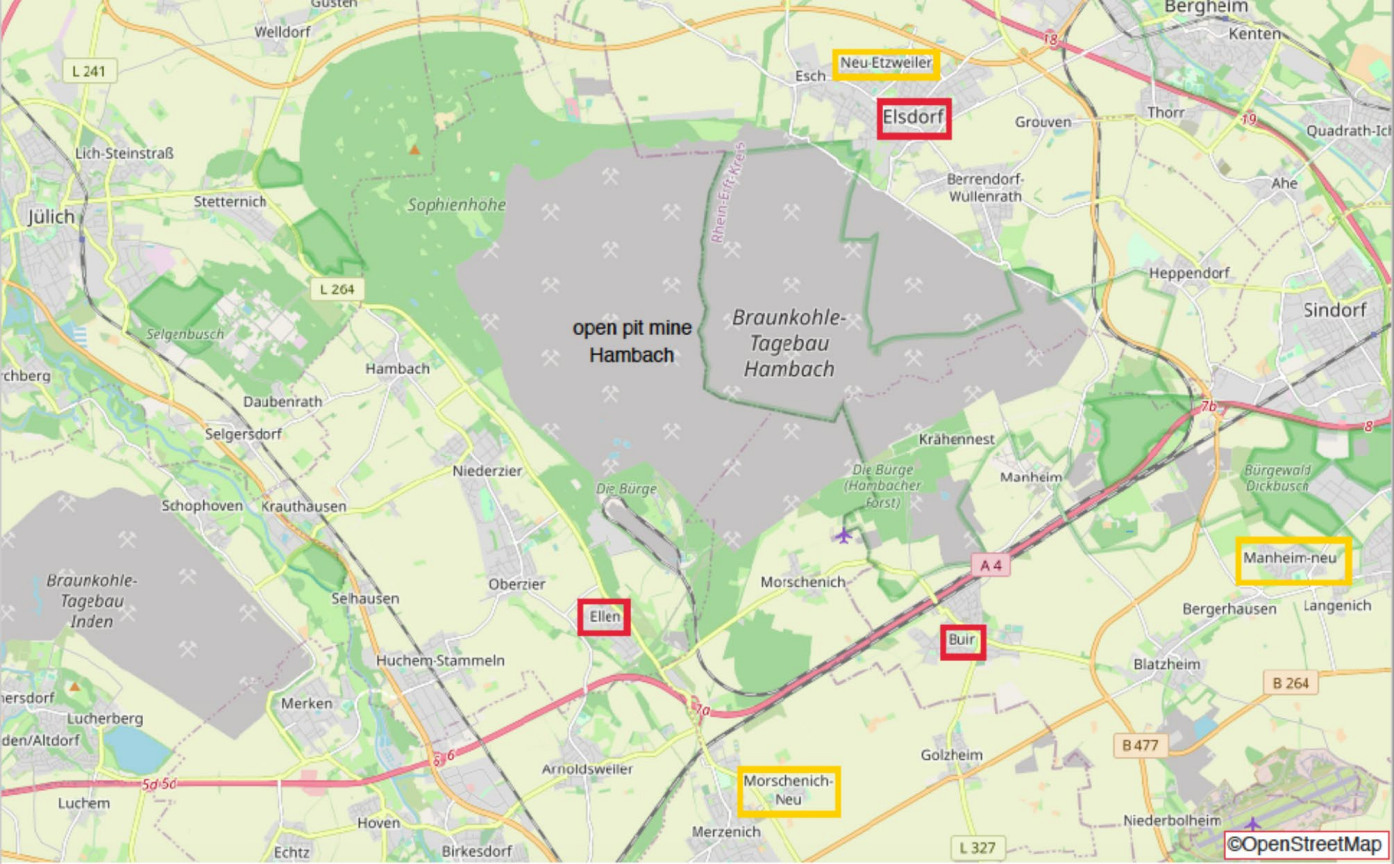
Map of the open pit mine Hambach and surroundings – places are marked (new villages - yellow; pit edge villages – red); OpenStreetMap contributors, edited by the authors.

The online questionnaire was implemented using SoSci Survey^35^ and made available to participants at www.soscisurvey.de. It was mandatory for the participants to specify the place of residence. Mainly participants from the Fig. 1 marked places answered the questionnaire. Other villages close to the open pit mine, such as Manheim or Morschenich were already completely abandoned, since the population was resettled.

### Questionnaire and survey scales/ measures

The questionnaire included 74 items for both groups, and an additional 24 items for the people in the new villages, resp. 14 for the people in the pit edge villages. Most questionnaire items were derived from a study by Krueger et al., which was prevailingly carried out at the neighboring open pit mine Garzweiler in the Rhenish region^14^. They mainly originate from the Environmental Distress Scale, developed in an Australian open pit mining area^15^. Krueger et al. translated the items into German and included six out of nine items (from the original scale used in the Australian indigenous population) based on local German conditions as well as interviews with residents^14,15^. Items were added based on literature research on the local context at the Hambach open pit mine. In addition, the Patient Health Questionnaire-9 & -15^36,37^ were used to determine potential mental burden.

First, sociodemographic data, such as age, gender, level of education and for participants from the new villages the period since the resettlement was completed. For all participants, the general effects of open pit mining, such as economic benefits, disagreements over the open pit mine in the community or family, destruction of buildings and nature were assessed. Those effects were rated using a five-point Likert scale from strongly agree to strongly disagree.

Only pit edge villagers were queried for experienced environmental hazards, such as dust, noise, vibration, or nocturnal lighting. Only new villagers were asked for stressors and protective factors during the resettlement process. This included questions concerning physical or psychological exhaustion, financial burden, living conditions, and place attachment to the old village. These questions used a 5-point Likert scale ranging from strongly agree to strongly disagree, as well.

All participants received questions about environmental and community actions including attended community meetings, demonstrations, petitions, or contact to politicians.

Concerning solastalgia, questions from the Australian solastalgia scale^15^ were used in accordance to other studies dealing with environmental damage^38,39^. The following questions were asked: “The sense of belonging undermined by mining-induced changes; “I am sad that native nature is being destroyed”; “I am worried that valued aspects of place – clean air, scenery- are being lost”; “I miss peace and quiet once enjoyed in this place”; “I feel sad when looking at mine voids and degraded landscapes”; “The farming lifestyle depending on good land and water is threatened by mining-induced changes”^40^. We used a 5-point Likert scale to measure the levels of solastalgic distress (i.e., 5 = strongly agree to 1 = strongly disagree), with 6 items and a total score ranking from 0 to 30, only evaluated when all items were answered.

Finally, the Patient Health Questionnaire (PHQ-9 & -15) were used in order to assess the mental burden of the participants. The Patient Health Questionnaires-9 & -15 (PHQ-9 & -15) are diagnostic tools for common mental health disorders that determine depressive and somatic symptoms, respectively^36,37^. They are widely used and validated especially in primary health care^41,42^. Symptoms were assessed as followed: 9 items for depressive symptoms during the last two weeks and 13 items for somatic symptoms within the last 4 weeks. PHQ-9 responses were ranked on a 4-point scale (0 = not at all, 1 = several days, 2 = more than half the days, 3 = nearly every day) with a total score from 0 to 27. A 3-point scale was used for the somatic symptoms (0 = not bothered at all, 1 = bothered a little, 2 = bothered a lot), with a total score from 0 to 30. According to the PHQ-D, the score for somatic symptoms is generated from the 13 items of the somatic symptom scale and 2 items of the depressive symptom scale. The two items of the PHQ-9 are scored as 0 (“not at all”), 1 (“on individual days”) and 2 (“on more than half of the days” or “almost every day”). Cut points of ≥ 5, ≥ 10, and ≥ 15 represent mild, moderate, and severe levels on both scales, respectively^43^.

For dichotomizing, we used a cut-off of ≥ 10, representing moderate-to-severe symptom levels. Not answered items were ranked as a 0, assuming that the symptoms did not occur.

### Statistical analyses

Data were analyzed using SAS Software, Version 9.4. Considering the cross-sectional design, descriptive statistical methods were used. Our main goal was to describe stressors, such as environmental stressors for the pit edge villagers and stressors caused by resettlement in the new village group, and to detect differences between our two study populations, the pit edge villagers, and the new villagers in terms of attitudes towards mining as well as the mental health burden. Therefore, we mainly used descriptive analyses of continuous and categorizable variables. We used in dependence of the variable, Chi-square or Kruskal-Wallis test in order to detect differences, for example concerning the mental burden between our two study populations. Gender-specific alterations were considered. The Spearman correlation coefficient was used for possible associations between the solastalgia-score, PHQ-scores, and period since completed resettlement. *p* < 0.05 was used as the level of significance.

## Results

**Fig. 2:**
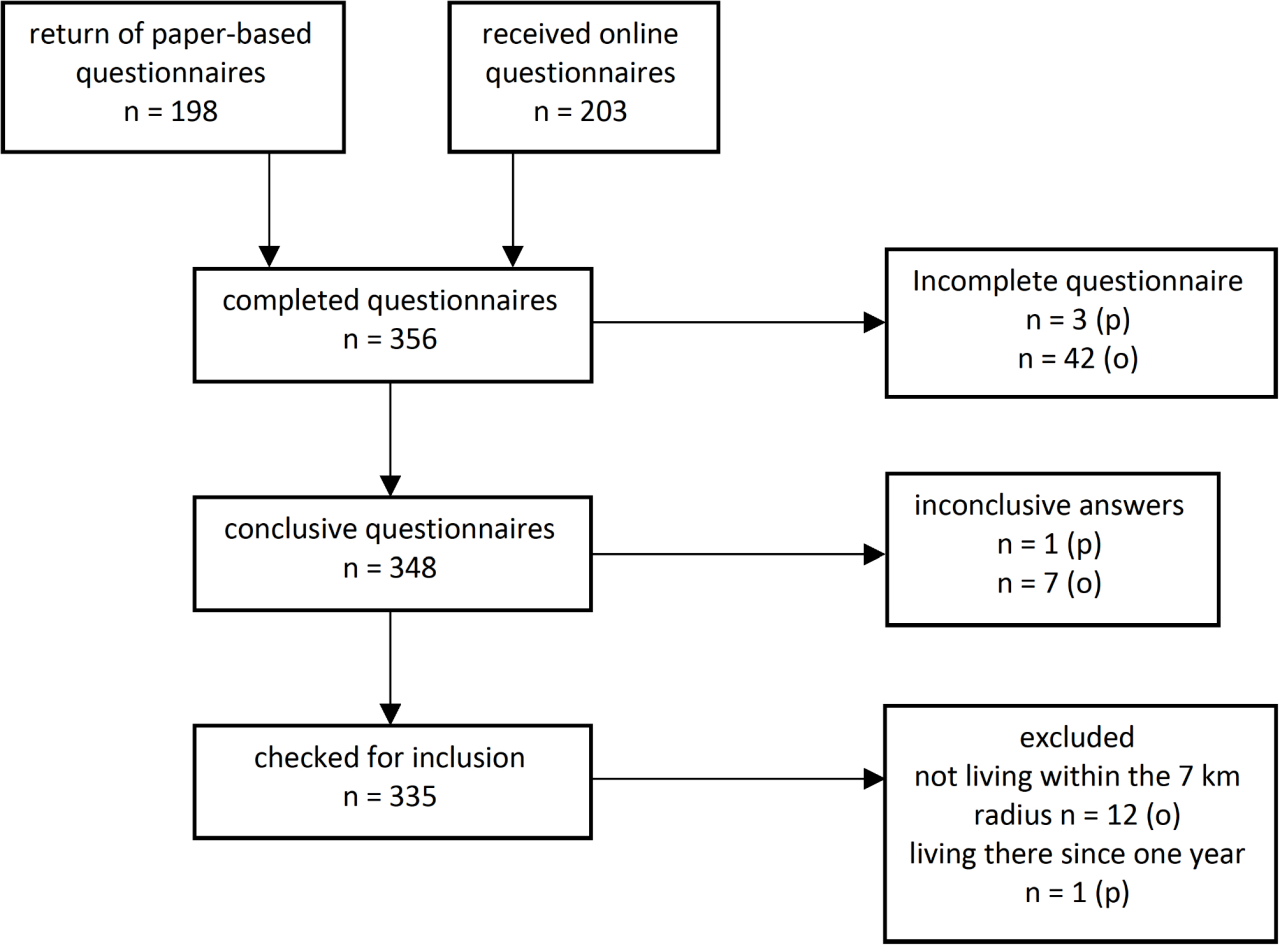
Flowchart of included and excluded participants.

A total of *n* = 335 questionnaires were included in the analyses, with *n* = 235 (70.1%) people living in pit edge villages and *n* = 100 (29.9%) people from new (resettled) villages. Questionnaires were excluded from the data analysis when people who lived outside of the 7 km radius next to the open pit mine, did not meet the minimum answer requirements or gave inconclusive answers. Included and excluded participants are shown Fig. 2.

### Sociodemographics

Table 1 gives the respondents’ sociodemographic characteristics. The average age of respondents in new villages was 57.1 years, in comparison to 53.6 years in pit edge villages. There were no significant differences for age, gender, marriage/ partnership, university degree, having children or grandchildren living in the village, ownership of residence, or having spent the entire lifetime in the village. 80.8% of the people in the new villages had former generations living in the region (vs. 61.8% in the pit edge villages; *p* < 0.001). Among respondents in new villages, also more people formerly lived on old family property (54.5% vs. 26.6%; *p* < 0.001).

The mean period since completed resettlement was 111.5 months (SD 100.5; with a range from 4 to 360 months) which is about 9.3 years (not shown in the Table). Excluding respondents who have lived in the village all their lives, the average length of residence of participants in pit edge villages is 27.7 years (not shown in the Table).

**Table 1:** Sociodemographics.

	New villages ^a^	Pit edge villages	*p*-Value*
	Mean (SD)		
Age	57.1 (17.0) *n* = 99	53.6 (15.6) *n* = 232	n.s.^#^
	*n* (%)		
Female gender	49 (49.0%) *n* = 100	114 (48.7%) *n* = 234	n.s.
Marriage or partnership	78 (78.0%) *n* = 100	182 (77.8%) *n* = 234	n.s.
University degree	21 (21.2%) *n* = 99	65 (27.8%) *n* = 234	n.s.
Children living in the same village ^b^	55 (55.0%) *n* = 100	107 (45.5%) *n* = 235	n.s.
Grandchildren living in the same village ^b^	9 (9.0%) *n* = 100	15 (6.4%) *n* = 235	n.s.
Former generations living in the region ^c^	80 (80.8%) *n* = 99	144 (61.8%) *n* = 233	< 0.001
Living on old family property	54 (54.5%) *n* = 99	62 (26.6%) *n* = 233	< 0.001
Ownership of residence	88 (88.9%) *n* = 99	213 (91.8%) *n* = 232	n.s.
Entire life spent in the village	43 (43.9%) *n* = 98	83 (36.2%) *n* = 229	n.s.

^a*^data refer to original location of residence (prior to resettlement), where relevant; ^b^ includes (grand)children living in the same household; ^c^ includes parents or older generations; * chi-square or (^#^) Kruskal-Wallis test; n.s. = not significant; SD = standard deviation.

### Solastalgia and the PHQ

Table 2 shows the scores of solastalgia, somatization, and depressive symptoms. The mean solastalgia score was slightly higher for people living at the pit edge of the open pit mine (18.8 vs. 18.3), though differences were not significant. Binary categorized by gender, female persons reached higher solastalgia scores (19.0 in new villages and 20.1 in pit edge villages) compared to male counterparts (17.5 in new villages and 17.4 in pit edge villages).

The PHQ scores were higher for respondents living in pit edge villages for both somatic (8.3 vs. 6.6, *p* < 0.05) and depressive symptoms (5.2 vs. 4.4), though differences were only significant for somatization. When gender-categorized, this significance remained in female respondents. In both scales and groups, female respondents always reached higher scores than male counterparts. Female persons living in pit edge villages showed the overall highest somatization (9.8 SD 6.2, *p* < 0.05) and depression scores (6.0, SD 5.5). Female persons reported also higher symptom severity after dichotomization in moderate-to-severe symptom levels. At least moderate levels of somatic symptoms were reported by 40.0% of respondents in pit edge villages (e.g., compared to 23.7% in new villages; *p* < 0.05). More than one in two female respondents from pit edge villages reported at least moderate somatic symptom levels, and thus twice as much as did female resettlers (50.5% vs. 25.0%; *p* < 0.05). In general, considerably less participants reported moderate-to-severe symptom levels for depression (12.8–20.6%) than they did for somatization (22.5–50.5%).

A moderate positive correlation between solastalgia and somatic symptoms (*r* = 0.45 for new villages and *r* = 0.51 for pit edge villages), respectively depressive symptoms (*r* = 0.46 for new villages and *r* = 0.52 for pit edge villages), was found (all *p* < 0.001), with a strong correlation in the group of the pit edge villages.

There was no clear correlation detected between the period since completed resettlement and solastalgia, depression, or somatic symptoms, respectively (data not shown).

**Table 2:** Solastalgia and Patient Health Questionnaire (PHQ) scores.

	New villages ^a^	Pit edge villages	*p*-Value*
	Solastalgia (score)		
	Mean (SD)		
Solastalgia	18.3 (7.8) *n* = 98	18.8 (7.6) *n* = 224	n.s.^#^
Male	17.5 (8.1) *n* = 50	17.4 (7.9) *n* = 110	n.s.^#^
Female	19.0 (7.4) *n* = 48	20.1 (7.2) *n* = 111	n.s.^#^
	PHQ (score)		
	Mean (SD)		
Somatization	6.6 (6.2) *n* = 93	8.3 (6.4) *n* = 213	< 0.05^#^
Male	6.2 (6.5) *n* = 49	6.9 (6.3) *n* = 103	n.s.^#^
Female	7.0 (5.8) *n* = 44	9.8 (6.2) *n* = 109	< 0.05^#^
Depression	4.4 (4.9) *n* = 91	5.2 (5.4) *n* = 210	n.s.^#^
Male	4.4 (5.2) *n* = 48	4.4 (5.2) *n* = 102	n.s.^#^
Female	4.5 (4.7) *n* = 43	6.0 (5.5) *n* = 107	n.s.^#^
	PHQ (dichotomized score > 9)		
	*n* (%)		
Somatization > 9	22 (23.7%) *n* = 93	85 (40.0%) *n* = 213	< 0.05
Male	11 (22.5%) *n* = 49	30 (29.1%) *n* = 103	n.s.
Female	11 (25.0%) *n* = 44	55 (50.5%) *n* = 109	< 0.05
Depression > 9	15 (16.5%) *n* = 91	35 (16.7%) *n* = 210	n.s.
Male	9 (18.8%) *n* = 48	13 (12.8%) *n =* 102	n.s.
Female	6 (14.0%) *n* = 43	22 (20.6%) *n* = 107	n.s.
	PHQ and Solastalgia (correlations)		
	r (*n*) *		
Somatization and solastalgia	0.45 (93)	0.51 (213)	< 0.001
Depression and solastalgia	0.46 (91)	0.52 (210)	< 0.001

^a^ data refers to original location of residence (prior to resettlement), where relevant; *chi-square or (^#^) Kruskal-Wallis test; n.s. = not significant; SD = standard deviation; r = Spearman correlation coefficient.

### Resettlement process and outcomes

65.9% of the resettled people indicated that their living conditions improved due to resettlement, see Table 3. Every second participant (49.5%) felt well informed/ advised by authorities during the resettlement process. Almost half (47.8%) of the people responded that they are looking ahead with a better feeling than before resettlement. On the other hand, 41.3% of the resettlers would have liked to spend the rest of their lives in their old village. Participants indicated a (strong) physical (33.0%) and psychological exhaustion (23.3%) due to the resettlement process. Two out of five respondents (41.1%) faced additional financial burden, while 28.3% said they lost contact with cherished people after relocation.

**Table 3:** Resettlement process and outcomes (responded by residents from new villages).

	*n* (%)*
Positive aspects	
Feeling more comfortable in new village	38 (41.8%) *n* = 91
Improved living conditions after resettlement	60 (65.9%) *n* = 91
Feeling well informed by authorities during resettlement process	45 (49.5%) *n* = 91
Looking ahead with a better feeling than before resettlement	44 (47.8%) *n* = 92
Negative aspects	
Feeling physically exhausted	30 (33.0%) *n* = 91
Feeling psychologically exhausted	21 (23.3%) *n* = 90
Extra financial burden	37 (41.1%) *n* = 90
Lost contact with cherished people	26 (28.3%) *n* = 92
Resisted the resettlement for a long time	25 (27.2%) *n* = 92
Would have liked to spend the rest of my life in my old village	38 (41.3%) *n* = 92

* % of answers with ‘strongly agree’ or ‘agree’.

### Impacts of and attitudes towards open pit mining

Table 4 shows positive and negative attitudes towards open pit mining. Over half of the resettlers (55.6%) indicated an understanding for open pit mining, in contrast to 43.0% of residents from pit edge villages. Less than half of both groups were satisfied with the authorities’ efforts to monitor the environmental impacts of open pit mining (42.4% in new villages vs. 47.3% in pit edge villages). 36.9% of the people from pit edge villages responded that they cannot/ could not enjoy life as much due to the open pit mine (vs. 33.0% of the people living in new villages). More than half of the respondents from pit edge villages (51.1%) were concerned that their health may be threatened by open pit mining in contrast to only 28.6% from the new villages. 52.0% of the resettlers (strongly) agreed on feeling ‘agitated/ angry about demonstrations’ (vs. 43.3%). In terms of place attachment (see Table 4), respondents from pit edge villages indicated a deeper connection to their village, a higher sense of responsibility for locals and a more than three times (63.4% vs. 18.7%) greater duty to maintain their place for future generations, compared to resettlers (when referring to their original place of residence).

**Table 4:** Attitudes towards open pit mining.

	New villages^#^	Pit edge villages
	*n* (%)	
Positive aspects		
Understanding for open pit mining	55 (55.6%) *n* = 99	98 (43.0%) *n* = 228
Economic advantages are important for the region	64 (65.3%) *n* = 98	130 (57.0%) *n* = 228
Financing of community projects by the mining company is helpful for the region	67 (67.7%) *n* = 99	143 (63.3%) *n* = 226
Satisfied with the efforts of the authorities to monitor the environmental impact of open pit mining	42 (42.4%) *n* = 99	104 (47.3%) *n* = 220
Negative aspects		
Could not enjoy my life as much through the open pit mining	33 (33.0%) *n* = 100	84 (36.9%) *n* = 228
Disagreements about open pit mine in		
Family	17 (17.0%) *n =* 100	23 (10.0%) *n =* 229
Village	30 (30.0%) *n =* 100	83 (36.6%) *n =* 227
Angry about destroyed		
Natural habitat for plants and animals	52 (52.5%) *n =* 99	133 (58.3%) *n* = 228
Historic buildings and landmarks	40 (40.0%) *n =* 100	120 (52.4%) *n* = 229
Effects of open pit mining are depressing	30 (30.6%) *n* = 98	76 (34.1%) *n* = 223
Agitated/ angry about demonstrations	51 (52.0%) *n* = 98	97 (43.3%) *n* = 224
Concerned that my health may be threatened by open-pit mining	28 (28.6%) *n* = 98	114 (51.1%) *n* = 223
Place attachment		
Feeling a deep connection to the place	41 (44.6%) *n* = 92	116 (54.4%) *n* = 213
Feeling a sense of responsibility for the people in the place	24 (26.4%) *n* = 91	103 (48.4%) *n* = 213
Feeling I have a duty to maintain the place for future generations	17 (18.7%) *n* = 91	135 (63.4%) *n* = 213

* % of answers with ‘strongly agree’ or ‘agree’; ^#^ data refer to original location of residence (prior to resettlement), where relevant.

### Environmental hazards

**Fig. 3:**
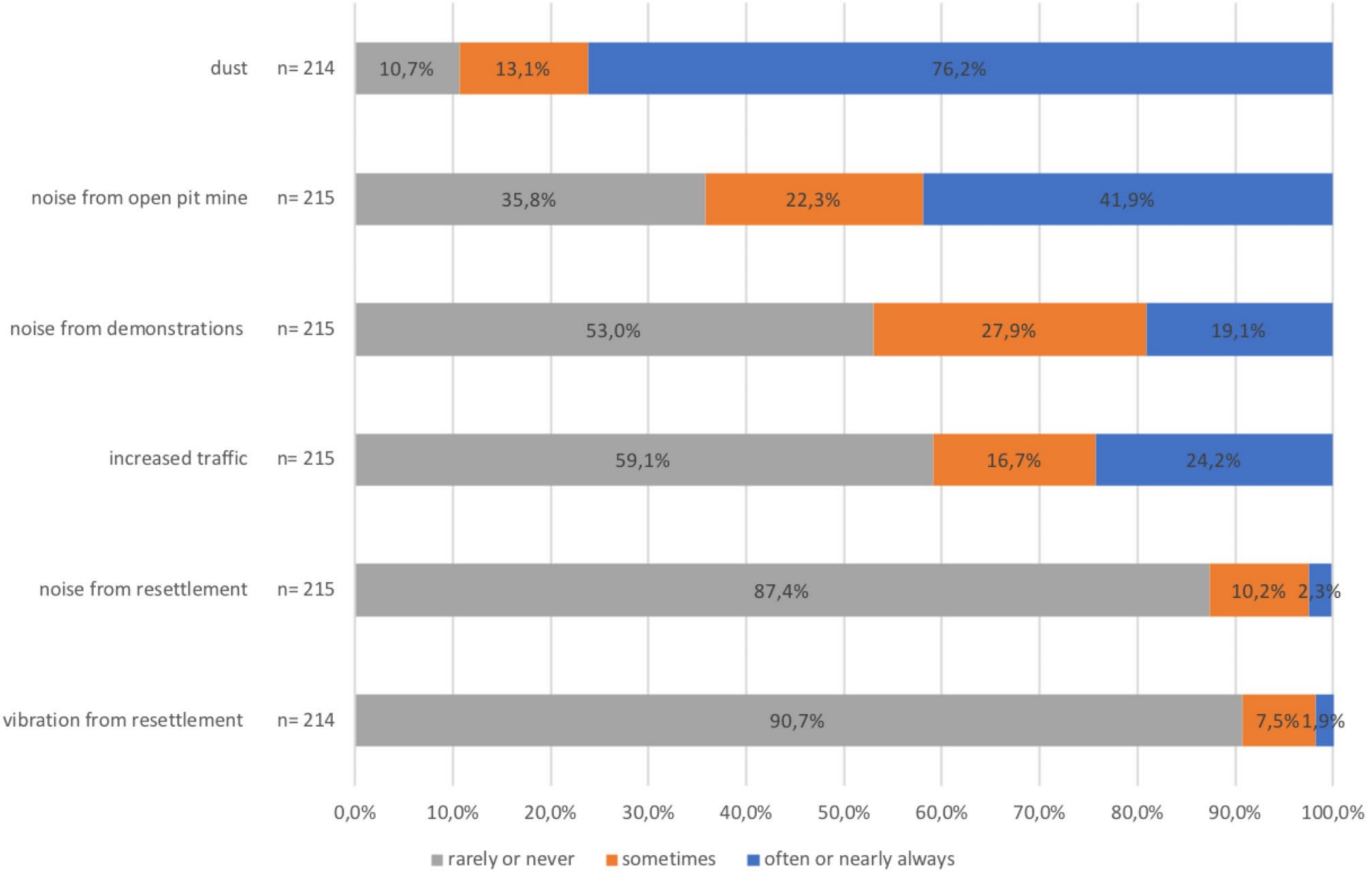
Frequency of experienced environmental hazards of respondents from pit edge villages.

Figure 3 lists potential environmental hazards related to open pit mining, responded by people living in pit edge villages. People felt most affected by dust (76.2% experienced it often to nearly always), followed by noise from the open pit mine (41.9%). Also, noise from demonstrations in the context of the open pit mine was experienced often to nearly always by one-fifth (19.1%) of respondents on a frequent basis. More than 85% of participants stated they were rarely or never affected by noise or vibration from resettlement activities.

### Environmental and community actions

**Fig. 4:**
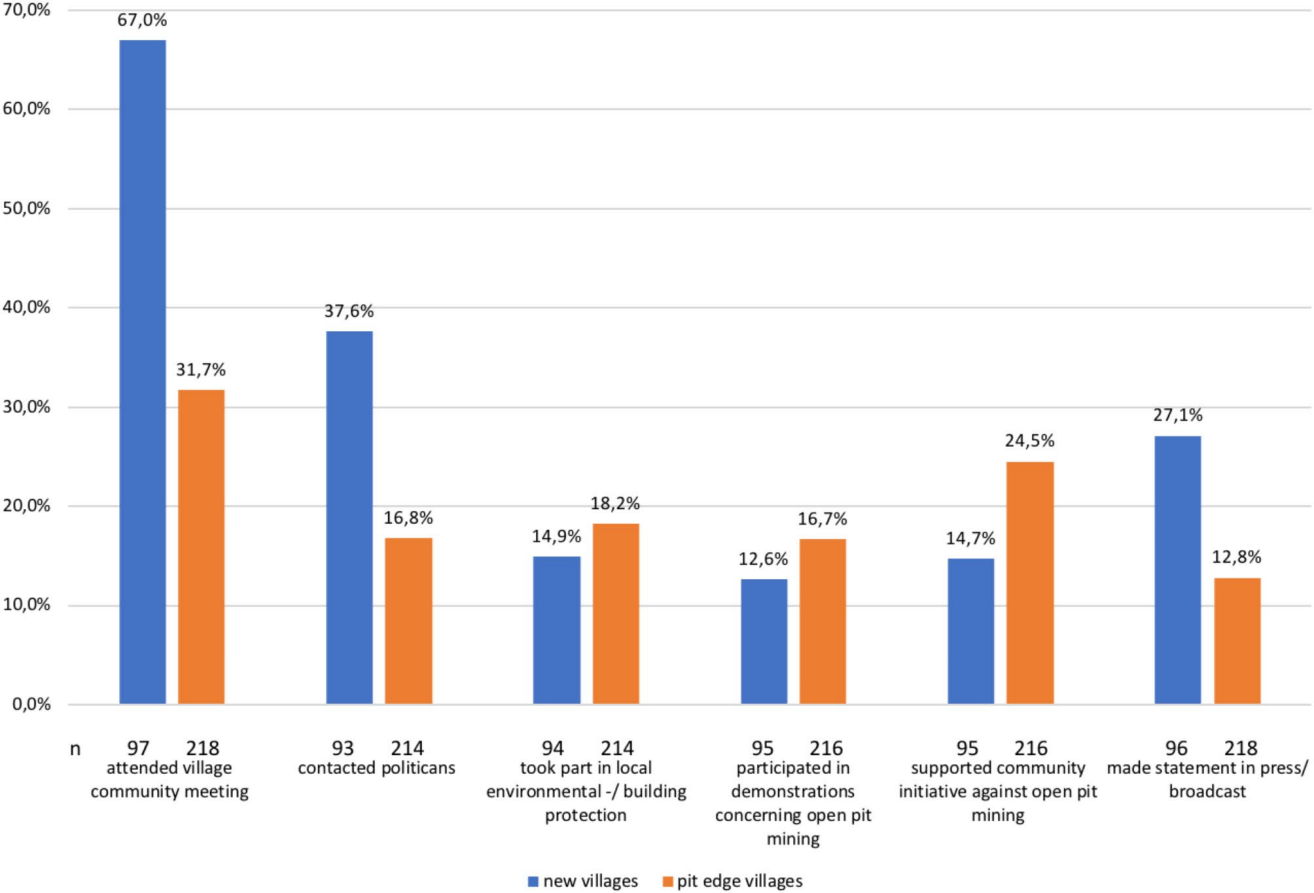
Environmental/ community actions that have been undertaken by the respondents split into the two groups.

The undertaken actions responding to the open pit mine vary between the groups of residents and resettlers (shown Fig. 4). For both groups, the most commonly reported activity was attending village community meetings, though more than twice as much among resettlers, compared to pit edge village respondents (67.0% vs. 31.7%). Frequently mentioned actions among people from new villages were contact to politicians (37.6%) and statements made in press/ broadcast (27.1%). Among pit edge village participants, the support of community initiatives against the open pit mine (24.5%) and taking part in local environmental or building protection measures (18.2%) were more common. Altogether, 48 participants (15.4%) declared having participated at least once in a demonstration against open pit mining.

### Free text comments

At the end of the questionnaire, respondents could annotate comments on the survey or elaborate on their personal situation. Just over 100 people took this opportunity. The following themes were emphasized or added on a frequent basis:

- Heavy exposure to dust from open pit mining.
- Sad feelings about the clearing and defragmentation of the Hambach Forest.
- Recultivated spoil pile (Sophienhöhe) – balanced positive and negating comments.
- Occupation of the forest by activists causes discomfort for some participants.
- Incomplete resettlement of one pit edge village led to anger and injustice.
- Additional mental burden through the COVID-19 pandemic and war in Ukraine.

## Discussion

Our study examined the living situation, mental health and specific stressor among residents and resettlers such as environmental degradation and the resettlement process associated with the open pit mine Hambach in Western Germany. Therefore, experienced effects of open pit mining and possible relocation, feelings of solastalgia and the presence of somatic and depressive symptoms were enquired. The mean scores of solastalgic, somatic and depressive symptoms as well as the frequency of moderate-to-severe symptom levels were higher among people still residing at the open pit mine in pit edge villages, compared to resettlers. Whether for depression, somatization or solastalgia, female respondents always reached higher scores than male counterparts. This trend, was already observed in previous studies^44–46^, even among open pit mining communities^47^ i.e. also in previous research in the Rhenish lignite region^14^.

Comparing the two groups, the sample size of the resettlers was significantly smaller. It should be noted that the population of people living in villages at the edge of the pit is much larger than the group of resettlers. In addition, the study was localized through the use of paper questionnaires and the local newspaper. Therefore, resettlers who have moved much further away may not have been reached by our survey, at all. In addition, some resettlers could have closed the matter with the move and do not want to be reminded of this for some quite emotional happening by taking part in surveys.

Comparing our findings with the general population, we found considerably increased levels in our both mining affected study groups:

According to the latest representative data in Germany from 2021, 8.8% of women and 7.5% of men in Germany reported at least moderate depressive symptoms (using the PHQ-8 questionnaire with a range of 0–24; cut-off score likewise ≥ 10)^48^. In our study cohorts, the values were higher, around twice as much for all (16.5 − 16.7%), as well as separated for females (20.6% − 14.0%) and males (18.8–12.8%). According to the latest data (2017) concerning the frequency of somatic symptoms in the general population, the mean PHQ-15 score (ranged from 0 to 28) was 5.5 (4.62 for males and 6.31 for females)^46^. Thus, in our study population scores where about 20% (new villages) to 50% (pit edge villages) higher (6.6 among resettlers and 8.8 in pit edge villages). These findings suggest that the observed association of open pit mining on the living environment and everyday life may be a potential psychological stress factor for local communities.

The concept of solastalgia originated in an Australian region that underwent increasing environmental impacts through open pit mining^33^. The meaning of solastalgia becomes more distinct, when looking at the more severe symptoms experienced by respondents from pit edge villages, i.e. who still live very close to the open pit mine. Solastalgic feelings refer to the ‘powerlessness’ experienced when your familiar surrounding environment changes, not when you change your surroundings (relocate)^33^. When communities remain living at their original home place, their relationship to that place may decline, when it loses its integrity^33^. The strong positive correlation between solastalgia and depressive or somatic symptoms found for open pit mine residents in this study underlines the psychological component within the concept of solastalgia. A strong feeling of solastalgia is therefore likely to contribute to the emergence or aggravation of mental disorders. Interestingly, no clear correlation was found between the period since completed resettlement and the respective symptom scores of solastalgia, depression and somatization, in the new villages group, similar to the study by Krueger in the Rhenish region^14^. One reason for this could be a too small study population of the resettlers. Otherwise, it suggests that “time does not heal all wounds” and psychological impairment may persist through the years. In light of this, the need for (long-term) psychosocial support services at community and individual level, seems even more necessary, to mitigate or prevent mental illness and improve general quality of life^46^.

Respondents from pit edge villages were stronger attached to their places (e.g., connection to place, responsibility for local people), than were resettlers to their original villages. The continuous alterations of the old place itself, resulting from the expanding open pit mine, resettlement activities and building demolitions, may reinforce the erosion of emotional memories and attachment for both groups. However, the data suggest that by changing location, the connection to the original place diminishes considerably. Higginbotham et al. already showed that people with a higher place attachment had stronger feelings of solastalgia, consistent with our data^15^. The importance of an established home becomes apparent by looking at the free text comments of our questionnaire. For participants who lived in the village of Morschenich and resettled before it was announced Morschenich will not to be demolished and the resettlements won’t be completed^49^, the main stated problem was the lacking possibility of reversing their resettlement by re-buying their old family home, but there are no clear sources regarding this topic. That new unknown people will live in their former traditional home, gave those resettlers a feeling of betrayal and injustice as described in our free text fields.

Mapping the current literature in Germany, Krueger et al. have already examined the mental health of communities at the neighboring open pit mine Garzweiler in the Rhenish region^14^. Although local and temporal circumstances differ in their study (e.g., resettlement process less advanced, with an additional third study group of individuals who were still to be resettled), the spatial proximity, similar sociodemographic characteristics and legal contexts of mining and resettlements, allows to draw comparisons: In line with our findings, higher scores of solastalgia, depression and somatization were found in the pit edge villages group, compared to new villages participants. There was also a similar trend in gender differences (female participants reported stronger psychological symptoms) and environmental hazards (dust as the most common problem). In general, the communities in proximity to the Garzweiler open pit mine reported higher scores in all psychological scales as well as solastalgia, than in this study. Taking into account the current conditions of the two locations, one explanatory approach for these differences lies within the time passed since completed resettlement. At the Hambach open pit mine, the resettlements took place years earlier and are now almost completed, whereas at the Garzweiler open pit mine they are a much more topical issue^50^. The latter could provoke more insecurity and psychological burden for all locals involved, explaining the higher symptoms levels at Garzweiler. Also, the ongoing resettlements at the open pit mine Garzweiler are associated with more activism on-site^51^. As seen in our study, the reactions of the residents to activism could also include feelings of stress, though this varies widely between respondents.

Also, the finding of our study in Germany cannot be generalized to open pit mines in other countries. Different local as well as cultural conditions need to be considered here. Further aspects influencing the well-being of affected populations could be sociodemographic factors, current recultivation measures and support systems, political situation (activism, groups who are still to be resettled) and pre-existing mental health status.

Dust, noise and increased traffic due to the open pit mine were the most frequently mentioned environmental hazards in this study. The estimation of the experienced frequency is naturally very subjective, depending on the person’s perception and vulnerability, exact place of residence, etc. Regardless, it has been proven that noise as well as (perceived) environmental pollution in general, can negatively impact the mental health of individuals^30,31^. For instance, Seidler et al. showed a positive relation between traffic noise and the risk of a newly diagnosed depression^11^. Taking the geographical location into account, the well-being of residents of the pit edge village Buir may also be affected by the proximity to the highway^12^, which is a source of noise and impaired air quality.

Most resettlers and pit edge village residents confirmed having attended a village community meeting concerning the open pit mining, though twice as much resettlers did so (67.0% vs. 31.7%). One reason for this could be that active participation in the resettlement process was supposedly intended by the companies/ authorities involved^52^, thus meeting spaces were presumably actively offered for those to be relocated. On the other hand, a will for a greater collective self-determination, cohesion and exchange during the resettlement seems plausible. In the process of relocation, it is important for the people concerned to make decisions about the location or infrastructure of the new village, in order to have some kind of control over their future and better deal with the consequences of environmental degradation^23,53^.

Regarding the resettlement process, only half of the resettlers (49.5%) felt well-informed by authorities. Access to information on planned procedures and available options during resettlement, as well as the role of autonomous decision-making, seem to be crucial for a successful relocation^53^.

Several limitations must be considered when interpreting our results. First, there is a huge difference in the number of participants of the two groups studied, with only *n* = 100 respondents from new villages. Due to the study design, and in particular by the use of an online questionnaire, no sufficient response rate can be estimated. Also, since the average resettlement was completed around 10 years ago, we cannot exclude recall bias. Further, PHQ-9 & -15 are self-reported, so there might be reporting biases, likely to rather overestimate the prevalence of depression or somatization^54^. Important to note, the PHQ-9 & -15 do not make definitive diagnoses of mental disorders. In addition, the questionnaire does not consider current events such as the COVID-19 pandemic or the conflict in Ukraine possibly amplifying symptoms of depression or somatization. Previous studies found that psychological stress, depression and anxiety symptoms increased since the beginning of the COVID-19 pandemic^55,56^. Our data collection took place in April and May 2022, when infection incidences and population-wide restrictions were low; nevertheless, the pandemic situation could have impacted our findings. Given that very recent population representative data (2021) on depression were available^48^, our results remain conclusive even under the influence of the COVID-19 pandemic. The recent war in Ukraine may have had a greater impact on the mental state on the general population, including our study group, but due to a lack of data precise effects cannot yet be estimated.

Mental health is influenced by further factors, including education, income, family and relationships, as well as physical health. In our cohort, although a high percentage of participants reported having university degree, a high PHQ symptom score was observed, although a higher education goes along with a lower likelihood of depressive symptoms^57^. Here, the belonging to the place and the influence of a changing environment could contribute to that fact. Physical health was not queried, a statement on the relation between physical and mental health can therefore not be derived in our cohort.

Due to the cross-sectional study design, no clear causal relationship between psychological distress and open pit mining can be inferred. The proximity of individual new villages to the open pit mine is also a matter of concern, as it could have an impact on their well-being. In addition, resettlers did not provide any information on potential environmental hazards in their new home. The questionnaire also may have missed relevant aspects, e.g., the profession and socioeconomic status (thus adaptability potential) of respondents. Especially in the vicinity of the open pit mine, occupational dependencies on the mining company cannot be ruled out and could have distorted our findings.

Participation in studies can be influenced by several variables. Data shows that an old age and a lower physical functioning has a negative impact on study participation^58^. This is particularly important as the group of resettlers includes more elderly, as relocation processes at the Hambach open pit mine have been carried out for a long time. Lower levels of income and a lower education makes it less likely that a person will complete the full questionnaire. Also, health problems are strong factors influencing participation in trials^59^. However, depression is not significantly related to study participation when examined alone^58^.

Furthermore, governmental efforts to mitigate the negative impacts of coal mining must also be taken into account as they can have an influence on attitudes towards open pit mining. To name just a few: financial assistance under the Structural Strengthening Act is to benefit local infrastructure in the affected region as well as renaturation and reforestation of former open pit mining areas^60^. Furthermore, innovative projects of communities, universities or companies are tried to be included in the design of the Rhineland area^61^.

The results of this study reveal a need for psychosocial support for local people affected by open pit mining and resettlement. In accordance with the recent findings of Kruger et al., the data from this study demonstrate higher symptom levels of psychological distress in residents who live or have lived right next to an open pit mine in comparison to the general population^14^. The elevated scores of those relocated, in this study often about a decade ago, also indicate that psychological distress, when present, can be long-term, extending beyond the period of active resettlement and familiarization with the new place. Especially for countries that still expand coal mining or other large-scale infrastructure projects, these findings give cause to early take care of the mental health of affected communities and to engage in dialogue with them. Extending communal or individual support services can be one possible undertaken measure, though the needs of those affected must be determined and respected according to their individual circumstances. Further research should focus on the resettlement processes themselves and how they can be made more socially and economically acceptable for local communities. Moreover, fair compensations for experienced environmental degradations in the familiar surroundings are considered helpful by the authors.

## Conclusions

The aim of our study was to assess environmental factors potentially affecting the mental health of people living in the vicinity of the Hambach open pit mine.

Both, respondents from pit edge villages and resettlers at the open pit mine Hambach, reported higher than usual depressive and somatic symptom levels and feelings of solastalgia, suggesting an impact of open pit mining and relocation on psychosocial health. Solastalgia, i.e. distress caused by a changing home environment, positively correlated with the symptom levels of depression and somatization. Respondents from pit edge villages felt most frequently affected by dust and noise derived from the open pit mine. 33% of the resettlers stated physically and 23.3% psychologically exhaustion due to the resettlement process. Only every second resident was satisfied with the authorities’ efforts to monitor environmental impacts of the open pit mine, while more than half of respondents from pit edge villages had health concerns.

Our data indicates the need for a dialogue with those affected by open pit mining processes including the health and well-being of residents and resettlers, respectively. Prospective studies to investigate causal relations between environmental changes and the mental health status need to be initiated. In addition, our results suggest an extension of implemented support services according to the individual circumstances.

Besides, the elaborated results can assist in developing further resettlement, recultivation and recompensation measures by taking the mental well-being of residents in proximity to open pit mines into account.

## Electronic supplementary material

Below is the link to the electronic supplementary material.

Supplementary Material 1

## Data Availability

The data that support the findings of this study are available from the corresponding author, Emma Holtermann, upon reasonable request.
