# Supplementary material for: Community perceptions and mental burden among (former) residents at Europe’s largest lignite mine in Western Germany: a cross-sectional study
Source: Sci Rep. 2025 Mar 14;15:8795. doi: 10.1038/s41598-025-92834-8 (PMC11909167; doi:10.1038/s41598-025-92834-8)
Supplement: Supplementary file 1 — Supplementary Material 1 [file 41598_2025_92834_MOESM1_ESM.docx]

**Solastalgia (Score)**

|  | New villages | Pit edge villages |
| --- | --- | --- |
|  | n (%* | |
| sense of belonging undermined by mining-induced changes | 26 (26,5%)  *n*= 98 | 37 (16,6%)  *n*= 223 |
| sad that native nature is being destroyed | 54 (55,1%)  *n*= 98 | 145 (64,7%)  *n*= 224 |
| worried that valued aspects of place—clean air, scenery—are being lost | 43 (43,9%)  *n*= 98 | 137 (61,4%)  *n*= 223 |
| miss peace and quiet once enjoyed in this place | 40 (40,8%)  *n*= 98 | 75 (33,5%)  *n*= 224 |
| sad when look at mine voids and degraded landscapes | 41 (41,8%)  *n*= 98 | 116 (51,8%)  n= 224 |
| farming lifestyle depending on good land and water is threatened by mining-induced changes | 37 (37,8%)  *n*= 98 | 104 (46,6%)  *n*= 223 |

**Table S1.** Items for calculating the solastalgia score

*% of answers with ‘strongly agree’ or ‘agree’

# Allgemeine Informationen zur Studie

**Worum geht es?** Im Rahmen meiner Doktorarbeit möchte ich untersuchen, inwiefern sich die Veränderungen von Heimat und Umwelt im Rheinischen Braunkohlerevier auf Gesundheit und Wohlbefinden der betroffenen Menschen auswirken.

**Teilnahmevoraussetzungen:** Sie sind mindestens 18 Jahre alt und aktuell in einer Ortschaft in der Nähe des Tagebaus Hambach wohnhaft oder waren vor Ihrer Umsiedlung in den vergangenen Jahren dort wohnhaft.

**Zeitdauer:** Die Bearbeitung dieses Fragebogens wird etwa 15 bis 20 Minuten dauern.

**Anonymität:** Alle Angaben, die Sie hier machen, werden anonym gespeichert und verarbeitet. Das bedeutet, dass zu keinem Zeitpunkt irgendwelche Angaben auf Sie persönlich zurückgeführt werden können.

**Kontakt:** Bei Fragen, Problemen oder Ähnlichem können Sie mich gern kontaktieren unter emma.holtermann@rwth-aachen.de oder 015774204913.

**Bei Nichtteilnahme entstehen für Sie keinerlei Nachteile!**

Mit dem Ausfüllen dieses Fragebogens bestätigen Sie, dass Sie die oben angegebenen Informationen gelesen und verstanden haben und mit der anonymen Verarbeitung Ihrer Daten einverstanden sind.

# Wichtige Hinweise zur Bearbeitung der Fragen

Verlassen Sie sich auf Ihre erste Reaktion und beantworten Sie die Fragen spontan.

Es gibt keine richtigen oder falschen Antworten. Bitte antworten Sie so, wie es für Sie am besten zutrifft. Bitte antworten Sie offen und ehrlich.

Bitte lassen Sie keine Frage aus. Sollten Sie einmal nicht die perfekt passende Antwort finden können, so wählen Sie bitte diejenige, die am ehesten auf Sie zutrifft.

Wenn Sie weiterführende Anmerkungen oder Rückmeldungen zum Fragebogen machen möchten, nutzen Sie dafür bitte das Freitextfeld auf der letzten Seite.

**Diese Studie schließt nur Personen ein, die in einer Ortschaft am Tagebau Hambach wohnhaft sind bzw. waren!**

**Wie alt sind Sie?**

…........... Jahre

**Welchem Geschlecht ordnen Sie sich zu?**

- männlich
- weiblich
- divers
- Ich möchte keine Angabe machen

**Welche Angabe trifft auf ihren aktuellen Familienstand zu?**

- ledig
- in einer Partnerschaft
- verheiratet
- verwitwet
- geschieden

**Welcher ist Ihr höchster Schulabschluss?**

- Haupt- oder Volksschulabschluss
- Realschule oder Polytechnische Hochschule
- Fachhochschulreife/ Abschluss einer Fachoberschule
- Abitur
- Schule beendet ohne Abschluss

**Welchen höchsten beruflichen Ausbildungs- bzw. Hochschulabschluss haben Sie?**

- Schüler:in
- noch in beruflicher Ausbildung (Auszubildene:r, Student:in, Berufsvorbereitungsjahr)
- keinen Berufsabschluss und nicht in beruflicher Ausbildung
- beruflich-betriebliche Ausbildung (Lehre) abgeschlossen
- beruflich-schulische Ausbildung (Berufsfachschule, Handelsschule) abgeschlossen
- Ausbildung an einer Fachschule, Meister-, Technikerschule, Berufs- oder Fachakademie abgeschlossen
- Bachelor an (Fach-) Hochschule abgeschlossen
- sonstiger Abschluss an einer Fachhochschule oder Ingenieurschule
- sonstiger Abschluss an einer Universität oder Hochschule
- anderer Bildungsabschluss

**Was trifft am ehesten auf Ihre momentane Wohnsituation zu?**

- Ich bin vor ………. Jahr(en) und ………. Monaten umgesiedelt und …
  - ich lebe in einer der Neusiedlungen (z.B. Neu-Etzweiler, Manheim-Neu, Morschenich-Neu)
  - ich lebe nicht in einer der Neusiedlungen
- Ich lebe in einer Ortschaft am Tagebau ohne Umsiedlerstatus (z.B. Niederzier, Merzenich)

1. Die folgenden Fragen beziehen sich auf das **Leben an** **Ihrer ursprünglichen Ortschaft**. Sollten Sie ggf. umgesiedelt sein, beziehen Sie sich bei der Beantwortung bitte auf Ihre vorherige Ortschaft.

**Wie lange leb(t)en Sie in Ihrer Ortschaft?**

- mein gesamtes Leben
- nicht mein gesamtes Leben, sondern …………. Jahre

**Wie viele Generationen Ihrer Familie lebten schon im Rheinischen Revier?**

- schon die Generation vor meinen Urgroßeltern
- meine Urgroßeltern
- meine Großeltern
- meine Eltern
- Ich bin in meiner Generation in die Region gezogen

**Leb(t)en Sie in einem Haus, einer Wohnung oder auf einem Grundstück, das von früheren Generationen Ihrer Familie bewohnt wurde?**

- ja
- nein

**Ich wohn(t)e in einem/r…**

- Haus/ Wohnung im eigenen oder familiären Besitz
- Haus/ Wohnung zur Miete

**Haben Sie Kinder? Wenn ja, wo leben diese?** (Mehrfachauswahl möglich)

- Ich habe Kinder,
  - die im gleichen Haushalt leben wie ich.
  - die in der gleichen Ortschaft leben wie ich.
  - die in der näheren Umgebung leben (<10 km).
  - die weiter entfernt leben (>10 km).
- Ich habe keine Kinder.

**Haben Sie Enkelkinder? Wenn ja, wo leben diese?** (Mehrfachauswahl möglich)

- Ich habe Enkelkinder,
  - die im gleichen Haushalt leben wie ich.
  - die in der gleichen Ortschaft leben wie ich.
  - die in der näheren Umgebung leben (<10 km).
  - die weiter entfernt leben (>10 km).
- Ich habe keine Enkelkinder.

1. Folgend finden Sie Aussagen zu den **persönlich empfundenen Auswirkungen des Tagebaus**. Bitte geben Sie an, inwieweit diese Aussagen für Sie zutreffen/nicht zutreffen.

|  | **trifft zu** | **trifft**  **eher zu** | **teils-teils** | **trifft eher**  **nicht zu** | **trifft nicht zu** |
| --- | --- | --- | --- | --- | --- |
| **Ich habe Verständnis für den Ausbau des Tagebaus.** |  |  |  |  |  |
| **Die wirtschaftlichen Vorteile des Tagebaus (z.B. Arbeitsplätze) für die Region sind wichtig.** |  |  |  |  |  |
| **Ich fühl(t)e mich durch den Tagebau nicht persönlich beeinträchtigt in …**  **… wirtschaftlicher Hinsicht**  **… gesundheitlicher Hinsicht** |  |  |  |  |  |
| **Ich kann/konnte das Leben wegen der Auswirkungen des Tagebaus nicht so sehr genießen, wie ich es gern möchte.** |  |  |  |  |  |
| **Meine Ortschaft ist/war durch Meinungsverschiedenheiten über den Tagebau gespalten.** |  |  |  |  |  |
| **Meine Familie ist/war durch**  **Meinungsverschiedenheiten über den Tagebau gespalten.** |  |  |  |  |  |
| **Ich bin verärgert über die Zerstörung von historischen Gebäuden und Wahrzeichen durch den Tagebau.** |  |  |  |  |  |
| **Ich bin verärgert über die Zerstörung von Wohnhäusern durch den Tagebau.** |  |  |  |  |  |
|  | **Trifft zu** | **Trifft eher zu** | **Teils-teils** | **Trifft eher nicht zu** | **Trifft nicht zu** |
| **Ich bin verärgert über die Zerstörung von natürlichem Lebensraum für Pflanzen und Tiere durch den Tagebau.** |  |  |  |  |  |
| **Es stört mich, dass bei der Erweiterung des Tagebaus künftige Generationen nicht mehr mitbedacht werden.** |  |  |  |  |  |
| **Die Finanzierung von Gemeinschafts- projekten durch das Tagebauunternehmen ist hilfreich für die Region.** |  |  |  |  |  |
| **Ich bin besorgt, dass meine Gesundheit durch den Tagebau bedroht sein könnte.** |  |  |  |  |  |
| **Ich fühle mich machtlos gegenüber den Veränderungen meiner Heimat.** |  |  |  |  |  |
| **Ich bin zufrieden mit den Bemühungen der Behörden, die Umweltfolgen des Tagebaus zu überwachen.** |  |  |  |  |  |
| **Die Auswirkungen des Tagebaus in der Region deprimieren mich.** |  |  |  |  |  |
| **Durch den Tagebau und die damit einhergehenden Veränderungen, wurde mein Gefühl, zu dieser Ortschaft zu gehören, beeinträchtigt.** |  |  |  |  |  |
| **Ich fühle mich aufgewühlt/ bin verärgert über die Demonstrationen.** |  |  |  |  |  |

|  | **trifft zu** | **trifft**  **eher zu** | **teils-teils** | **trifft eher**  **nicht zu** | **trifft nicht zu** |
| --- | --- | --- | --- | --- | --- |
| **Ich bin traurig, dass durch den Tagebau heimische Natur zerstört wird.** |  |  |  |  |  |
| **Ich bin besorgt, dass Aspekte dieses**  **Ortes, die ich schätze, verloren gehen**  **(z.B. saubere Luft, schöne Landschaften)** |  |  |  |  |  |
|  | **Trifft zu** | **Trifft eher zu** | **Teils-teils** | **Trifft eher nicht zu** | **Trifft nicht zu** |
| **Ich vermisse das Gefühl von Frieden und Ruhe, das ich früher einmal in dieser Ortschaft genossen habe.** |  |  |  |  |  |
| **Ich bin traurig, wenn ich Tagebaulöcher und zerstörte Landschaften betrachte.** |  |  |  |  |  |
| **Ich fürchte, dass durch den Tagebau die regionale Landwirtschaft geschädigt wird.** |  |  |  |  |  |

1. Bitte geben Sie an, ob Sie eine der genannten **Aktivitäten als Reaktion auf die Auswirkungen**

**des Tagebaus** in Ihrer Region durchgeführt haben.

|  | **ja** | **nein** |
| --- | --- | --- |
| **Meinungsäußerung in Presse oder Rundfunk getätigt** |  |  |
| **Teilnahme an einem Treffen der Dorfgemeinschaft zur Diskussion der Tagebauauswirkungen** |  |  |
| **Ich stand aufgrund meiner Bedenken zu den Auswirkungen des Tagebaus in Kontakt mit**  **… politischen Amtsinhaber:innen**  **… rechtlichen Vertreter:innen oder Institutionen**  **… Umweltbehörden oder -verbänden**  **… dem Tagebauunternehmen** |  |  |
| **Petition gegen den Ausbau des Tagebaus unterschrieben** |  |  |
| **Teilgenommen an lokalen Umwelt- oder Gebäudeschutzmaßnahmen** |  |  |
| **Teilgenommen an Demonstrationen gegen den Tagebau** |  |  |
| **Unterstützung einer Bürgerinitiative, die sich gegen den Ausbau des Tagebaus einsetzt** |  |  |

1. Die folgenden Aussagen richten sich nur an Teilnehmende, die eine **Umsiedlung bereits vollständig abgeschlossen** haben.

**Alle anderen Teilnehmenden gehen bitte weiter zu Box 5.**

Bitte geben Sie an, inwieweit diese Aussagen für Sie zutreffen/ nicht zutreffen.

|  | **trifft zu** | **trifft**  **eher zu** | **teils-**  **teils** | **trifft eher**  **nicht zu** | **trifft nicht zu** |
| --- | --- | --- | --- | --- | --- |
| **In meiner neuen Ortschaft fühle ich mich wohler als in meiner alten Ortschaft.** |  |  |  |  |  |
| **Ich blicke mit einem besseren Gefühl in die Zukunft als vor der Umsiedlung.** |  |  |  |  |  |
| **Ich hätte mein weiteres Leben gern in meiner alten Ortschaft verbracht.** |  |  |  |  |  |
| **Ich fühle noch eine tiefe Verbundenheit zu meiner alten Ortschaft.** |  |  |  |  |  |
| **Ich habe mich innerlich lange gegen die Umsiedlung gewehrt.** |  |  |  |  |  |
| **Ich fühle mich weiterhin für die Menschen in meiner alten Ortschaft verantwortlich.** |  |  |  |  |  |
| **Ich wünsche mir, dass meine alte Ortschaft noch für zukünftige Generationen erhalten bleibt.** |  |  |  |  |  |
| **Ich fühle mich verpflichtet, meine alte Ortschaft noch für zukünftige Generationen zu erhalten.** |  |  |  |  |  |
| **Durch die Umsiedlungen habe ich Kontakt zu mir liebgewonnenen Menschen verloren.** |  |  |  |  |  |
| **Die Umsiedlung belastet mich psychisch oder hat mich psychisch belastet.** |  |  |  |  |  |
| **Ich fühle oder fühlte mich durch die Umsiedlung körperlich erschöpft.** |  |  |  |  |  |
| **Die Umsiedlung ging für mich mit einer finanziellen Mehrbelastung einher.** |  |  |  |  |  |
| **Meine berufliche Situation hat sich durch die Umsiedlung verschlechtert.** |  |  |  |  |  |
| **Die Ausübung meiner Freizeitaktivitäten hat sich durch die Umsiedlung verschlechtert**  **(z.B. Vereinsaktivität, Sport, Spaziergänge).** |  |  |  |  |  |
|  | **Trifft zu** | **Trifft eher zu** | **Teils-teils** | **Trifft eher nicht zu** | **Trifft nicht zu** |
| **Durch die Umsiedlung haben sich meine allgemeinen Lebensbedingungen verbessert (z.B. altersgerechtes Wohnen oder nahe Einkaufsmöglichkeiten)** |  |  |  |  |  |
| **Während des Umsiedlungsprozesses fühlte ich mich von den verantwortlichen Stellen gut informiert, beraten und unterstützt.** |  |  |  |  |  |
| **Es belastet mich, dass meine alte Ortschaft vielleicht erhalten bleibt und nicht abgerissen wird.** |  |  |  |  |  |
| **Die Gemeinschaft in der neuen Ortschaft zeigt einen stärkeren Zusammenhalt als vor der Umsiedlung.** |  |  |  |  |  |
| **Ich fühle mich in der neuen Ortschaft mehr durch die Umwelt belastet als in meiner alten Ortschaft (z.B. durch Baulärm).** |  |  |  |  |  |

|  | **trifft zu** | **trifft**  **eher zu** | **teils-**  **teils** | **trifft eher**  **nicht zu** | **trifft nicht zu** |
| --- | --- | --- | --- | --- | --- |
| Bitte beantworten Sie die Frage nur, wenn Sie Nutztiere besitzen:  **Meine Nutztiere kann ich in der neuen**  **Ortschaft genauso gut halten wie früher.** |  |  |  |  |  |

|  | **ja** | **nein** | **keine**  **Angabe** |
| --- | --- | --- | --- |
| **Die Umsiedlung ging für mich mit dem Verlust von Grundstücksfläche oder Land einher.** |  |  |  |
| Bitte beantworten Sie die Frage nur, wenn Sie Nutztiere besitzen:  **Vor der Umsiedlung besaßen ich und meine Familie mehr Nutztiere.** |  |  |  |

1. Die folgenden Aussagen richten sich nur an Teilnehmende, die in einer **Ortschaft ohne Umsiedlerstatus** am Tagebau leben, also nicht von Umsiedlung betroffen sind.

**Alle anderen Teilnehmenden gehen bitte weiter zu Box 6.**

Bitte geben Sie an, inwieweit diese Aussagen für sie zutreffen/ nicht zutreffen.

|  | **trifft zu** | **trifft**  **eher zu** | **teils-**  **teils** | **trifft eher**  **nicht zu** | **trifft nicht zu** |
| --- | --- | --- | --- | --- | --- |
| **Ich fühle eine tiefe Verbundenheit** **zu dieser Ortschaft.** |  |  |  |  |  |
| **Ich empfinde eine gewisse Verantwortung für die Menschen an dieser Ortschaft.** |  |  |  |  |  |
| **Ich fühle mich verpflichtet, meine Ortschaft noch für zukünftige Generationen zu erhalten.** |  |  |  |  |  |
| **Die Veränderungen durch den Tagebau beeinflussen meine Lebenssituation negativ.** |  |  |  |  |  |
| **Aufgrund der Veränderungen durch den Tagebau würde ich meine Ortschaft verlassen, wenn ich könnte.** |  |  |  |  |  |

Folgend finden Sie Umgebungsbelastungen durch den Tagebau, die Sie in Ihrer Ortschaft möglicherweise erleben bzw. erlebt haben. Bitte geben Sie an, wie oft Sie diese erleben bzw. erlebt haben.

|  | **nie** | **selten** | **manchmal** | **oft** | **fast immer** |
| --- | --- | --- | --- | --- | --- |
| **Staub (z.B. Kohlestaub)** |  |  |  |  |  |
| **Lärm durch Tagebauaktivitäten (z.B. Lastwagen)** |  |  |  |  |  |
| **Vibration oder Erschütterung durch**  **Tagebauaktivitäten**  **(z.B. Schaufelradbagger)** |  |  |  |  |  |
| **Lärm durch Umsiedlungen (z.B. Häuserabriss)** |  |  |  |  |  |
| **Vibration oder Erschütterung durch Umsiedlungen (z.B. Häuserabriss)** |  |  |  |  |  |
|  | **Nie** | **Manchmal** | **Selten** | **Oft** | **Fast immer** |
| **Nächtliche Lichtbelästigung durch Tagebauaktivitäten** |  |  |  |  |  |
| **Erhöhtes Verkehrsaufkommen (z.B.**  **durch Umleitungen)** |  |  |  |  |  |
| **Lärm durch Demonstrationen (z.B. Hubschrauber)** |  |  |  |  |  |

|  | **ja** | **nein** | **keine**  **Angabe** |
| --- | --- | --- | --- |
| **Bergschäden an ihrer/m Wohnung/Haus oder Grundstück** |  |  |  |

1. Die folgenden Aussagen richten sich an **alle Teilnehmenden**.

Bitte geben Sie an, wie stark Sie sich im Verlauf der letzten **4 Wochen** durch die folgenden **Beschwerden** beeinträchtigt gefühlt haben.

|  | **Nicht beeinträchtigt** | **Wenig beeinträchtigt** | **Stark beeinträchtigt** |
| --- | --- | --- | --- |
| **Bauchschmerzen** |  |  |  |
| **Rückenschmerzen** |  |  |  |
| **Schmerzen in den Armen, Beinen oder Gelenken (Knie, Hüften usw.)** |  |  |  |
| Falls zutreffend: **Menstruationsschmerzen oder andere Probleme bei der Menstruation** |  |  |  |
| **Schmerzen oder Probleme beim Geschlechtsverkehr** |  |  |  |
| **Kopfschmerzen** |  |  |  |
| **Schmerzen im Brustbereich** |  |  |  |
| **Schwindel** |  |  |  |
| **Ohnmachtsanfälle** |  |  |  |
| **Herzklopfen oder Herzrasen** |  |  |  |
| **Kurzatmigkeit** |  |  |  |
| **Verstopfung, nervöser Darm oder Durchfall** |  |  |  |
|  | **Nicht beeinträchtigt** | **Wenig beeinträchtigt** | **Stark beeinträchtigt** |
| **Übelkeit, Blähungen oder Verdauungsbeschwerden** |  |  |  |

1. Die folgenden Aussagen richten sich an **alle Teilnehmenden**.

Bitte geben Sie an, wie oft Sie sich im Verlauf der letzten **2 Wochen** durch die folgenden **Beschwerden** beeinträchtigt gefühlt haben.

|  | **Überhaupt nicht** | **An einzelnen**  **Tagen** | **An mehr als der Hälfte der Tage** | **Beinahe jeden Tag** |
| --- | --- | --- | --- | --- |
| **Nervosität, Ängstlichkeit oder Anspannung** |  |  |  |  |
| **Nicht in der Lage sein, Sorgen zu stoppen oder zu kontrollieren** |  |  |  |  |
| **Übermäßige Sorgen bezüglich verschiedener Angelegenheiten** |  |  |  |  |
| **Schwierigkeiten zu entspannen** |  |  |  |  |
| **Rastlosigkeit, so dass Stillsitzen schwer fällt** |  |  |  |  |
| **Schnelle Verärgerung oder Gereiztheit** |  |  |  |  |
| **Gefühl der Angst, so als würde etwas Schlimmes passieren** |  |  |  |  |
| **Wenig Interesse / Freude an Ihren Tätigkeiten** |  |  |  |  |
| **Niedergeschlagenheit, Schwermut oder Hoffnungslosigkeit** |  |  |  |  |
| **Schwierigkeiten, ein- oder durchzuschlafen, oder vermehrter Schlaf** |  |  |  |  |
| **Müdigkeit oder das Gefühl, keine Energie mehr zu haben** |  |  |  |  |
| **Verminderter Appetit oder das übermäßige Bedürfnis zu essen** |  |  |  |  |
| **Schlechte Meinung von sich selbst; Gefühl ein Versager zu sein oder die Familie enttäuscht zu haben** |  |  |  |  |
|  | **Überhaupt nicht** | **An einzelnen Tagen** | **An mehr als der Hälfte der Tage** | **Beinahe jeden Tag** |
| **Schwierigkeiten, sich auf etwas zu**  **konzentrieren, z.B. beim Zeitungslesen oder Fernsehen** |  |  |  |  |
| **Waren Ihre Bewegungen und Ihre Sprache so verlangsamt, dass es auch anderen auffallen würde? Oder waren Sie im Gegenteil „zappelig“ oder ruhelos und hatten dadurch einen stärkeren Bewegungsdrang als sonst?** |  |  |  |  |
| **Gedanken, dass Sie lieber tot wären oder sich Leid zufügen möchten** |  |  |  |  |

1. Bitte nutzen Sie für weiterführende **Anmerkungen, Erklärungen oder Kommentare** zu Ihrer persönlichen Situation oder zum Fragebogen das folgende Freitextfeld (optional).

|  |
| --- |

Sollten Sie beim Ausfüllen dieses Fragebogens bemerkt haben, dass Sie sich durch die Auswirkungen des Tagebaus und Veränderungen Ihrer Heimat gesundheitlich stark belastet fühlen und möglicherweise professionelle Unterstützung benötigen, wenden Sie sich bitte an Dr. Andrea Kaifie- Pechmann (Institut für Arbeits-, Sozial und Umweltmedizin, Uniklinik RWTH Aachen) zur weiteren Beratung: akaifie@ukaachen.de oder 0241 / 8035345.

**Vielen Dank für Ihre Teilnahme an dieser Studie!**

**Bitte senden Sie den Fragebogen innerhalb von zwei Wochen** **in dem vorfrankierten Umschlag zurück an:**

PD Dr. med. Andrea Kaifie-Pechmann und Emma Holtermann

Institut für Arbeits-, Sozial- und Umweltmedizin

Uniklinikum RWTH Aachen

Pauwelsstraße 30 52074 Aachen
